# EuroSCORE or regional system for cardiac operative risk evaluation?

**DOI:** 10.1186/1749-8090-8-S1-O261

**Published:** 2013-09-11

**Authors:** B Mihajlovic, L Velicki, S Nicin, S Susak, BB Mihajlovic, M Fabri

**Affiliations:** 1 Cardiovascular Surgery, Institute of Cardiovascular Diseases Vojvodina, Serbia; 2 Cardiology, Institute of Cardiovascular Diseases Vojvodina, Serbia

## Background

During the last decade many authors find that European Systems for Cardiac Operative Risk Evaluation (EuroSCORE), additive and logistic models, overestimate risk in cardiac surgery. The new EuroSCORE II has been recently introduced as an update to previous versions. The aim of the study: to investigate the prognostic value and discriminatory power of the additive and logistic EuroSCORE, and the EuroSCORE II.

## Methods

The research included 1247 consecutive patients who underwent cardiac surgery (718 coronary, 294 valvular and 233 combined), from January 2012 to March 2013 at our institute. The predicted mortality according to the additive EuroSCORE, logistic EuroSCORE and the new EuroSCORE II were compared with observed mortality-30 days after surgery.

## Results

The observed mortality rate was 3.45%, while the predicted mortality, regarding the additive model, logistic model and the EuroSCORE II was 4.43%, 5.27% and 2.12%, respectively. The difference between predicted and observed mortality was statistically significant for the logistic EuroSCORE (p=0.002) and the EuroSCORE II (p=0.004), but it was not statistically significant for the additive EuroSCORE (p=0.092). The areas under the ROC curves were statistically different from 0.5 for all three models (additive model AUROC=0.754; 95%CI(0.684 - 0.827), logistic model AUROC=0.759, 95%CI(0.688 - 0.830), EuroSCORE II AUROC=0.743; 95%CI(0.666 - 0.883)). Sensitivity: the additive model 69.8%; the logistic model 67.4%; the EuroSCORE II 72.1%. Specificity: the additive model 68.9%; the logistic model 75.9%; the EuroSCORE II 71.2%.

## Conclusion

In our population, the additive and logistic EuroSCORE overestimate risk in cardiac surgery. However, the EuroSCORE II, significantly underestimates operative risk. The discriminary power of all three models is not satisfactory. We believe that the regionally derived model would faithfully illustrate the actual state of patient population of the region where it was developed.

